# CRISPR deletion of the *C9ORF72* promoter in ALS/FTD patient motor neurons abolishes production of dipeptide repeat proteins and rescues neurodegeneration

**DOI:** 10.1007/s00401-020-02154-6

**Published:** 2020-04-07

**Authors:** Gopinath Krishnan, Yu Zhang, Yuanzheng Gu, Mark W. Kankel, Fen-Biao Gao, Sandra Almeida

**Affiliations:** 1 Department of Neurology, University of Massachusetts Medical School, Worcester, MA 01605 USA; 2 Department of Neurology, Xinhua Hospital Affiliated to Shanghai Jiao Tong University School of Medicine, Shanghai, China; 3 Neuromuscular and Movement Disorders, Biogen, Cambridge, MA 02142 USA

GGGGCC (G_4_C_2_) repeat expansion in the first intron of *C9ORF72* is the most common genetic cause of amyotrophic lateral sclerosis (ALS) and frontotemporal dementia (FTD) [6, 11]. Brain tissues from affected individuals show characteristic nuclear RNA foci containing the expanded repeat RNAs, as well as neuronal inclusions containing dipeptide repeat (DPR) proteins [poly(GA), poly(GP), poly(GR), poly(PR), and poly(PA)] resulting from the translation of both sense and antisense repeat RNAs in all reading frames [4, 9, 14]. Although reduced C9ORF72 protein function may contribute to disease [10], the more likely drivers of disease are mechanisms related to a gain of toxic function [7]. Currently, intense efforts are being made to identify disease mechanisms amenable for the development of therapeutic strategies. One promising avenue would be to prevent the production of the expanded repeat RNAs, such as by antisense oligonucleotides [5]. Here, we tested another potential therapeutic approach: CRISPR/Cas9-based targeting of the promoter region.

In ALS and FTD patients, transcription initiated at exon-1a of *C9ORF72* generates RNA species containing G_4_C_2_ repeat expansions that are in turn translated into three DPR proteins, poly(GA), poly(GP), and poly(GR). Thus, we first used reporter constructs to identify the promoter sequence 5′ to exon-1a that is responsible for transcription initiation of expanded repeat RNA. We cloned the 435 nucleotides (nt) upstream of the predicted transcription initiation site, as well as several truncated fragments of the 435-nt sequence, into a luciferase reporter vector (Fig. 1a) and expressed all the constructs in HEK293 cells. Deletion of the first 301 nt did not reduce the relative expression of the reporter gene. However, deleting the remaining 134 nt abolished luciferase expression (Fig. 1a), indicating that the core promoter elements are located in this region. To further characterize this sequence, we also tested a construct lacking the first 394 nt and one lacking the remaining 41 nt. Each of these deletions reduced the relative luciferase expression, indicating that both constructs lacked a portion of sequence required for reporter gene expression (Fig. 1a). These experiments indicate that the last 134 nt sequence contains the minimal promoter necessary to drive expression of G_4_C_2_ repeat-containing *C9ORF72* transcripts.

**Fig. 1:**
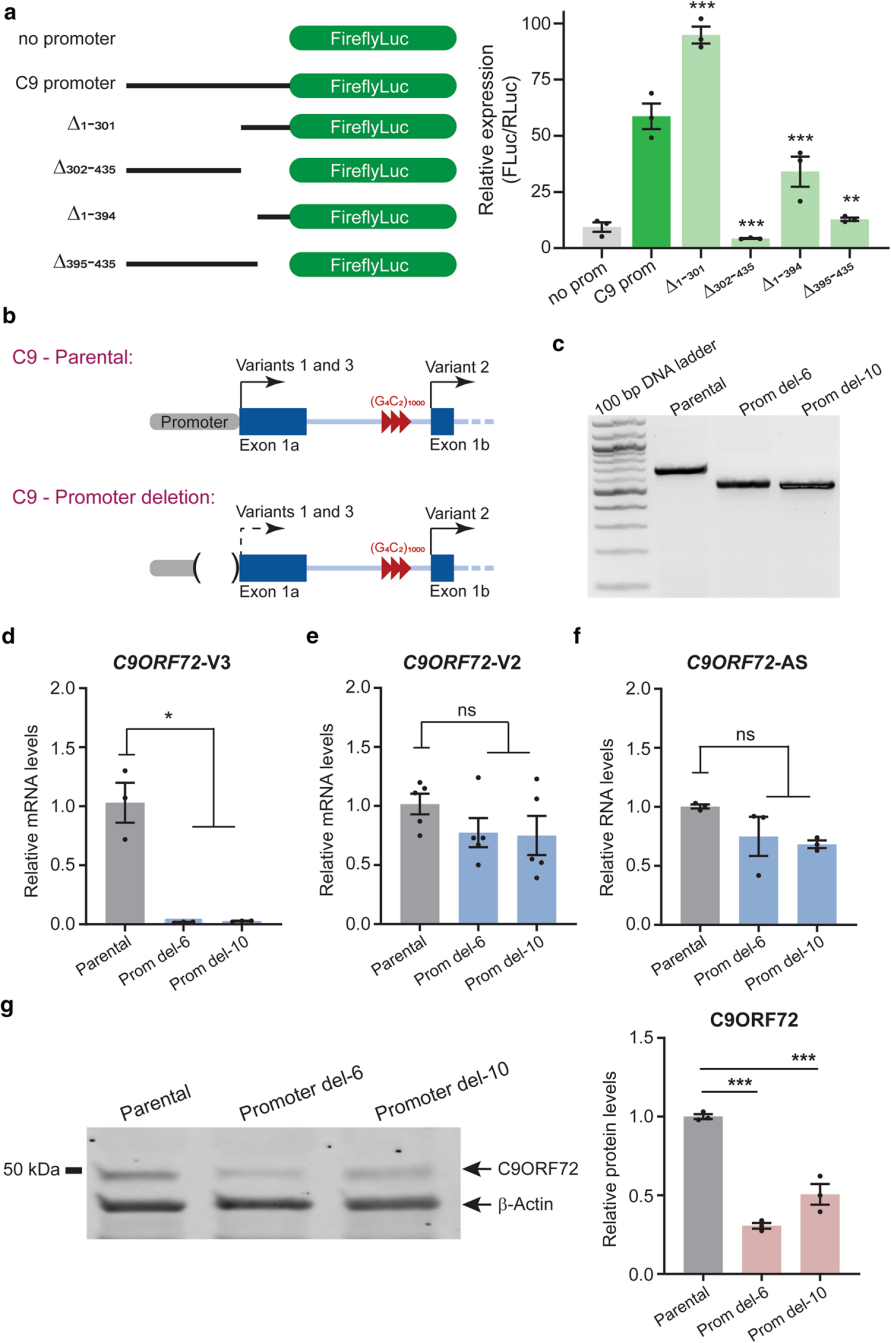
Deletion of the *C9ORF72* promoter region. **a** Schematic of the different promoter deletions analyzed in the luciferase reporter assay and quantification of the relative expression of firefly luciferase (FLuc)/renilla luciferase (RLuc) in HEK293 cells (*n* = 3 independent transfections). **b** Schematic of the *C9ORF72* locus in parental and promoter deletion iPSC-derived neurons. Arrows indicate the site of transcription initiation for the different variants. Only variants containing exon 1a are expected to be affected by the deletion; the variant containing exon 1b is predicted to use a different promoter. **c** CRISPR-edited iPSC lines are homozygous for the 140-bp deletion as indicated by the PCR analysis. **d–f** Four-week-old motor neurons from parental and promoter deletion lines were analyzed for expression of *C9ORF72*-V3, *C9ORF72*-V2, and *C9ORF72*-antisense RNAs (*n* = 3 independent differentiations). **g** C9ORF72 protein levels in 4-week-old parental and promoter deletion motor neurons (*n* = 3 independent differentiations). Values are mean ± SEM. *p < 0.05, **p < 0.01, ***p < 0.001 (**a**, **e–g**, one-way ANOVA; **d**, Welch’s *t* test). *ns* not significant

To investigate how this deletion affects the production of RNAs containing G_4_C_2_ repeat expansions in *C9ORF72* human neurons, we used CRISPR-Cas9 technology to generate a similar deletion 5′ to exon-1a of *C9ORF72* in an induced pluripotent stem cell (iPSC) line containing ~ 1000 copies of the G_4_C_2_ repeats [2] (Figs. 1b, c; S1). We selected two iPSC lines containing the promoter deletion and differentiated them and the parental iPSC line into ChAT-positive motor neurons (see Suppl. Information). As expected, the promoter deletion eliminated the expression of *C9ORF72*-Variant 3 (V3), whose transcription starts on exon-1a (Fig. 1b, d). In contrast, the RNA level of *C9ORF72*-V2, whose translation starts on exon 1b, or the expression of *C9ORF72*-antisense RNA, was not significantly affected (Fig. 1e–f), consistent with the fact that these transcripts have their own promoters [6]. In iPSCs and motor neurons harboring the promoter deletion, we detected a reduction in the levels of full-length C9ORF72 protein (Figs. 1g; S2), likely due to loss of *C9ORF72*-V3 expression.

Since RNAs containing the G_4_C_2_ repeat expansion lead to the production of DPR proteins, we next measured DPR protein levels in parental and promoter deletion motor neuron cultures using Meso Scale Discovery immunoassays. We found that expression of poly(GA), poly(GP), and poly(GR) was almost completely abolished in the motor neuron cultures harboring the promoter deletion (Fig. 2a–c). We then examined the functional consequence of reducing DPR levels in these neurons, using axonal degeneration as our scoring metric as we reported recently [12]. Deletion of the promoter region prevented the increase in axonal degeneration of parental motor neurons upon withdrawal of neurotrophic factors for 2 weeks (Figs. 2d, e; S3), a phenotype we previously described in neurons differentiated from two pairs of isogenic *C9ORF72* iPSC lines [12]. The promoter deletion also prevented upregulation of the expression of the heat shock protein *HSPA1B* mRNA (Fig. 2f), a phenotype detected in ALS/FTD patient brain tissues and *C9ORF72* iPSC-derived motor neurons [3, 8].

**Fig. 2:**
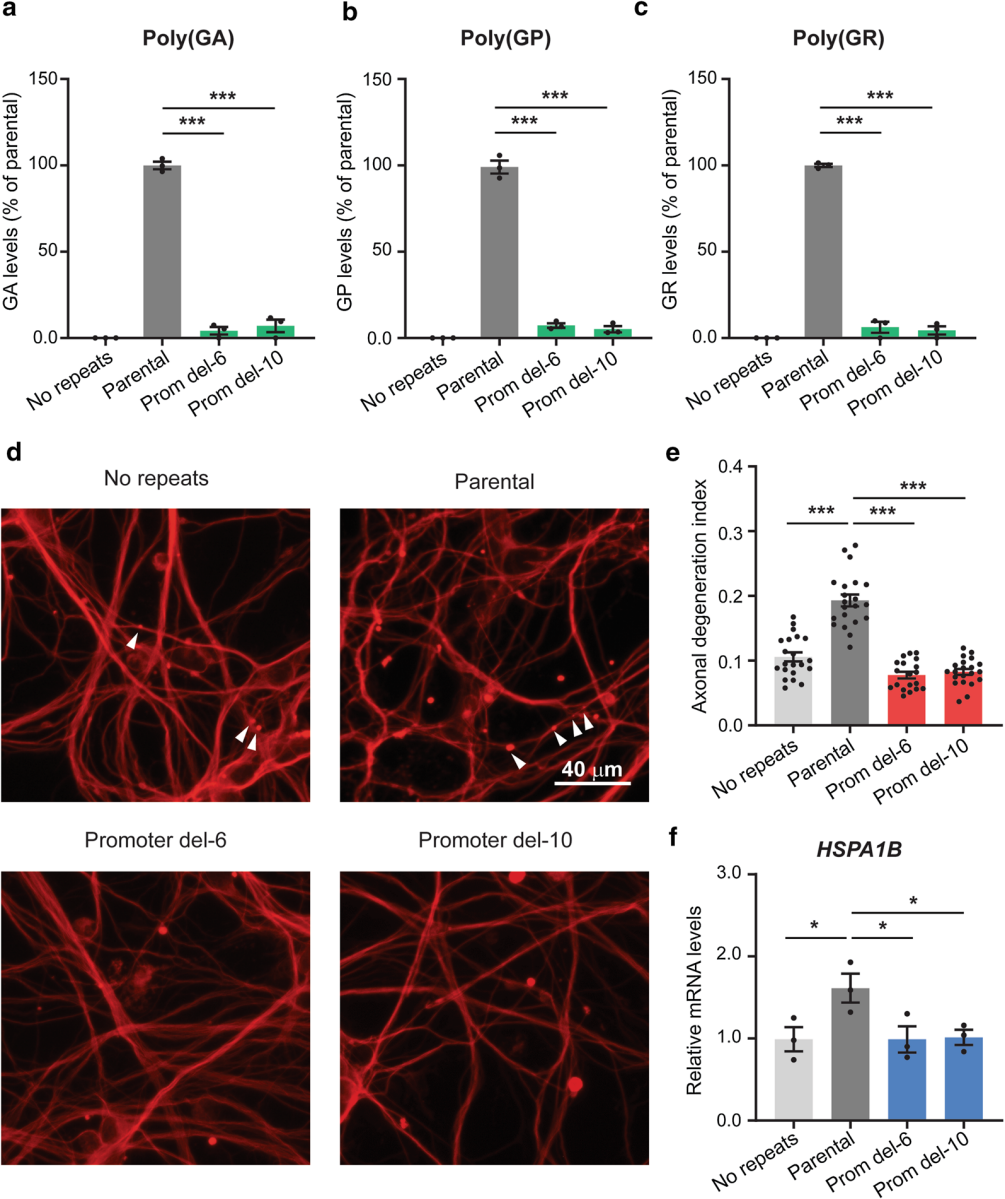
Effects of *C9ORF72* promoter deletion in iPSC-derived neurons. **a**–**c** Poly(GA), poly(GP), and poly(GR) levels in 1.5-month-old parental neurons, neurons without repeats, and promoter deletion motor neurons (*n* = 3 independent differentiations) were measured with Meso Scale Discovery immunoassays. The poly(GA) assay was done in a blinded manner at Biogen. **d** Representative immunofluorescence images of the axonal degeneration assay done with the marker βΙΙΙ-tubulin (TUJ1^+^), which revealed swollen varicosities and axonal fragments. Arrowheads indicate fragmented axons. **e** Axonal degeneration assessed by measuring the ratio of fragmented axons over the total TUJ1^+^ area 2 weeks after withdrawal of neurotrophic factors. Six to eight randomly selected fields were analyzed for each condition and each neuronal culture. Each independent data point represents one field, (*n* = 3 independent differentiations). **f** Relative expression of *HSPA1B* in 10-week-old motor neuron cultures (*n* = 3 independent differentiations). Values are mean ± SEM. *p < 0.05, ***p < 0.001 (one-way ANOVA, Dunnett’s multiple comparisons test)

Our results indicate that deletion of the 134 nt minimal promoter 5′ to exon-1a in *C9ORF72* prevents both the production of sense RNAs containing expanded G_4_C_2_ repeats and the activation of downstream neurodegeneration pathways. These findings also suggest that sense repeat RNA and its translation products are responsible for the observed neurodegenerative phenotypes, at least in this experimental system. The promoter deletion also partially decreased C9ORF72 protein levels. However, in the absence of DPR proteins, loss of C9ORF72 does not cause neurodegeneration in mice or iPSC-derived motor neurons [1, 10]. Improved versions of the CRISPR interference are now being tested in vivo to silence gene transcription without cutting genomic DNA [13]. Thus, our proof-of-concept study suggests that CRISPR/Cas9-based targeting of the promoter region to eliminate sense repeat RNA and its toxic translation products may be a potentially useful therapeutic approach for *C9ORF72*-ALS/FTD, especially before significant accumulation of DPR proteins.

## Electronic supplementary material

Below is the link to the electronic supplementary material.

Supplementary file1 (PDF 807 kb)
