# Supplementary material for: CRISPR deletion of the C9ORF72 promoter in ALS/FTD patient motor neurons abolishes production of dipeptide repeat proteins and rescues neurodegeneration
Source: Acta Neuropathol. 2020 Apr 7;140(1):81–4. doi: 10.1007/s00401-020-02154-6 (PMC7300081; doi:10.1007/s00401-020-02154-6)
Supplement: Supplementary file 1 — Supplementary file1 (PDF 807 kb) [file 401_2020_2154_MOESM1_ESM.pdf]

Supplementary Information

A. Supplementary figures

Parental (exon 1a/b in gray)

AAACAAAACCATTCAAAACCACGAAATCGTCTTCACTTTCTCCAGATCCAGCAGCCTCCCCTATTAAGGTTCGCACA
CGCTATTGCGCCAACGCTCCTCCAGAGCGGGTCTTAAGATAAAAGAACAGGACAAGTTGCCCCGCCCCATTTCGCT
AGCCTCGTGAGAAAACGTCATCGCACATAGAAAACAGACAGACGTAACCTACGGTGTCCCGCTAGGAAAGAGAG
GTGCGTCAAACAGCGACAAGTTCCGCCCACGTAAAAGATGACGCTTGGTGTGTCAGCCGTCCCTGCTGCCCGGTT
GCTTCTCTTTTGGGGGCGGGGTCTAGCAAGAGCAGGTGTGGGTTTAGGAGGTGTGTGTTTTTGTTTTTCCCACCCT
CTCTCCCCACTACTTGCTCTCACAGTACTCGCTGAGGGTGAACAAGAAAAGACCTGATAAAGATTAACCAGAAGAA
AACAAGGAGGGAAACAACCGCAGCCTGTAGCAAGCTCTGGAACTCAGGAGTCGCGCGCTA(GGGGCC)$_{\sim 1,000}$GGG
GCGTGGTCGGGGCGGGCCCGGGGGCGGGCCCGGGGCGGGGCTGCGGTTGCGGTGCCTGCGCCCGCGGCGGCG
GAGGCGCAGGCGGTGGCGAGTGG

Promoter deletion – 6: 140-bp deletion

AAACAAAACCATTCAAAACCACGAAATCGTCTTCACTTTCTCCAGATCCAGCAGCCTCCC - - - - - - - - - - - - - - - - - -
- -
- -CCTACGGTGTCCCGCTAGGAAAGAGAG
GTGCGTCAAACAGCGACAAGTTCCGCCCACGTAAAAGATGACGCTTGGTGTGTCAGCCGTCCCTGCTGCCCGGTT
GCTTCTCTTTTGGGGGCGGGGTCTAGCAAGAGCAGGTGTGGGTTTAGGAGGTGTGTGTTTTTGTTTTTCCCACCCT
CTCTCCCCACTACTTGCTCTCACAGTACTCGCTGAGGGTGAACAAGAAAAGACCTGATAAAGATTAACCAGAAGAA
AACAAGGAGGGAAACAACCGCAGCCTGTAGCAAGCTCTGGAACTCAGGAGTCGCGCGCTA(GGGGCC)$_{\sim 1,000}$GGG
GCGTGGTCGGGGCGGGCCCGGGGGCGGGCCCGGGGCGGGGCTGCGGTTGCGGTGCCTGCGCCCGCGGCGGCG
GAGGCGCAGGCGGTGGCGAGTGG

Promoter deletion – 10: 141-bp deletion

AAACAAAACCATTCAAAACCACGAAATCGTCTTCACTTTCTCCAGATCCAGCAGCCTCCC - - - - - - - - - - - - - - - - - -
- -
- -CTACGGTGTCCCGCTAGGAAAGAGAG
GTGCGTCAAACAGCGACAAGTTCCGCCCACGTAAAAGATGACGCTTGGTGTGTCAGCCGTCCCTGCTGCCCGGTT
GCTTCTCTTTTGGGGGCGGGGTCTAGCAAGAGCAGGTGTGGGTTTAGGAGGTGTGTGTTTTTGTTTTTCCCACCCT
CTCTCCCCACTACTTGCTCTCACAGTACTCGCTGAGGGTGAACAAGAAAAGACCTGATAAAGATTAACCAGAAGAA
AACAAGGAGGGAAACAACCGCAGCCTGTAGCAAGCTCTGGAACTCAGGAGTCGCGCGCTA(GGGGCC)$_{\sim 1,000}$GGG
GCGTGGTCGGGGCGGGCCCGGGGGCGGGCCCGGGGCGGGGCTGCGGTTGCGGTGCCTGCGCCCGCGGCGGCG
GAGGCGCAGGCGGTGGCGAGTGG

Supplementary Fig. 1 Sequencing data from each of the *C9ORF72* promoter deletion iPSC lines and the parental line.

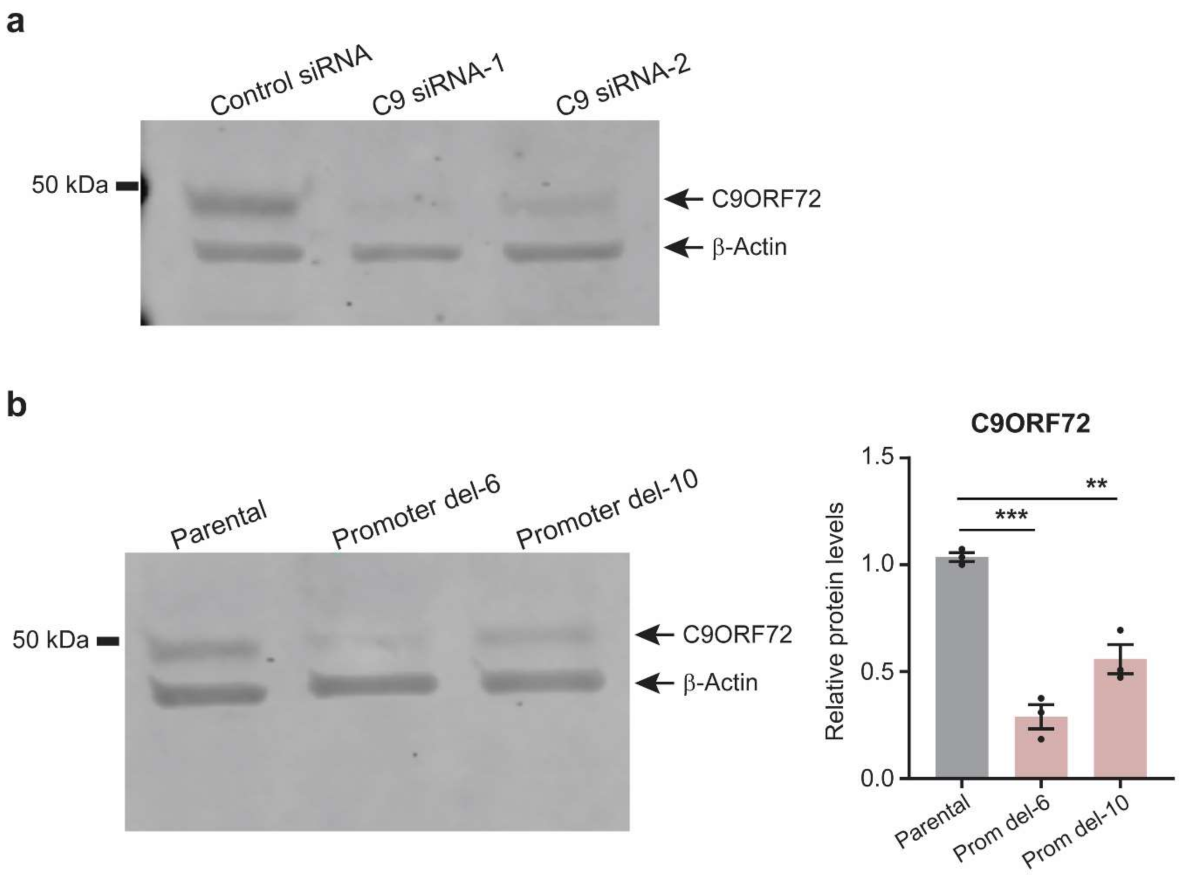

Supplementary Fig. 2 a To confirm the specificity of the C9ORF72 antibody (GeneTex, Cat. no. GTX634482), HEK293 cells were transfected with control or *C9ORF72* siRNAs (Thermo Fisher Scientific, Cat. no. 4392420, siRNA-ID#s4790, siRNA-ID#s4791), and C9ORF72 protein levels were analyzed by western blotting. **b** C9ORF72 protein levels in parental and promoter deletion iPSCs ($n = 3$ independent cultures). Values are mean ± s.e.m. $**p < 0.01$; $***p < 0.001$ (one-way ANOVA, Dunnett's multiple comparisons test).

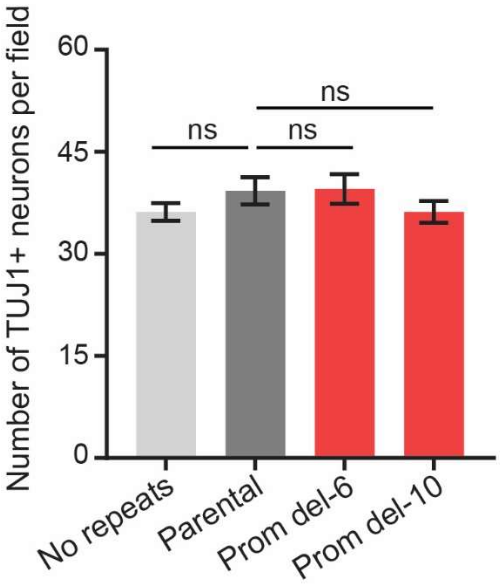

Supplementary Fig. 3 Quantification of the number of TUJ1+ neurons per field for the axonal degeneration assay. Six to eight randomly selected fields were analyzed for each condition and each neuronal culture. Values are mean ± s.e.m. for 3 independent differentiations. ns, not significant.

B. Supplementary methods

Generation of *C9ORF72* promoter deletion iPSC lines. Genome editing with the CRISPR-Cas9 system to create a deletion in the first intron of *C9ORF72,* 5′ to the G_4C_2 repeats was done at ALSTEM (Richmond, CA). Briefly, the Neon electroporation system was used to transfect iPSCs with two guide RNAs: TGTGCGAACCTTAATAGGGG and AAACAGACAGACGTAACCTA. Single cells were placed in 96-well plates, cultured for 14 days, and expanded. Genomic DNA from each clone was extracted with the Zymo genomic extraction kit. Clones with the desired homozygous deletion were identified with a PCR amplification assay, and the PCR products were sent for sequencing. iPSCs were expanded and collected to isolate genomic DNA. The region of interest was amplified by PCR, and the products were sent for sequencing to confirm the identity of each clone (Supplementary Fig. 1). The no-repeats iPSC line was generated and characterized previously (Lopez-Gonzalez et al., 2019). The deletion carried by this line eliminated the approximately 1000 G_4C_2 repeats present in the original *C9ORF72* parental line.

Motor neuron cultures. Motor neurons were differentiated as described before (Lopez-Gonzalez et al., 2016). Briefly, iPSC colonies were seeded on Matrigel-coated wells in mTeSR1 medium (StemCell Technologies); 24 h later, the medium was changed to neuroepithelial progenitor (NEP) medium [1:1 DMEM/F12:Neurobasal, 0.5x N2, 0.5x B27, 0.1 mM ascorbic acid (Sigma), 1x Glutamax, 3 μM CHIR99021 (StemCell Technologies), 2 μM DMH1 (StemCell Technologies), and 2 μM SB431542 (Stemgent)] and replaced every other day for 6 days. Progenitor colonies were dissociated with Accutase, seeded at 1:6 on Matrigel-coated wells, and cultured in NEP medium containing 0.1 μM retinoic acid and 0.5 μM purmorphamine for 6 days; the medium was replaced every other day. Motor neuron progenitors were lifted, cultured in suspension for 6 additional days in the absence of CHIR99021, DMH1, and SB431542, and dissociated to single cells with Accutase. Cells were seeded on poly-lysine/laminin-coated wells in motor neuron medium (1:1 DMEM/F12:Neurobasal, 0.5x N2, 0.5x B27, 0.1 mM ascorbic acid, 1x Glutamax, 10

ng/ml BDNF, 10 ng/ml GDNF, 1 μg/mL laminin, 0.1 μM Compound E, 0.5 μM retinoic acid, and 0.1 μM purmorphamine) for up to 10 weeks. For the axonal degeneration assay, the neurons were cultured for 2 weeks in the same neuron medium to which BDNF and GDNF were not added. This protocol generated a culture with >90% ChAT-positive neurons. All experiments were done with neurons derived from 3 independent differentiations.

Measurement of poly(GR) and poly(GP). Poly(GR) and poly(GP) were measured with a immunoassay as described (Choi et al., 2019). Briefly, neurons were lysed in ice-cold RIPA buffer (Thermo Fisher Scientific) or Tris-lysis buffer (Meso Scale Discovery) containing a cocktail of protease and phosphatase inhibitors (Thermo Fisher Scientific), sonicated on ice at a 20% pulse rate for 15 sec, and centrifuged at 16,000 *g* for 20 min at 4°C. The protein content of supernatants was determined with the BCA assay reagent (Thermo Fisher Scientific). Neurons samples (2 μg/l) were loaded on a 96-well single-spot plate (Meso Scale Discovery; Cat. no. L45XA) pre-coated with a custom-made polyclonal rabbit anti-$(GR)_8$ or anti-$(GP)_8$ antibodies (1 μg/ml, Covance) and tested in duplicate wells. Serial dilutions of recombinant $(GR)_8$ or $(GP)_8$ peptide in 1% BSA-TBST were used to prepare the standard curve. The detection antibodies were anti-$(GR)_8$ or anti-$(GP)_8$ antibodies previously tagged with GOLD SULFO (GOLD SULFO-TAG NHS-Ester Conjugation Pack, Meso Scale Discovery; Cat. no. R31AA) at a concentration of 0.5 μg/ml. Response signals from the assay plate were acquired with a QuickPlex SQ120 instrument (Meso Scale Discovery). For background correction, values from a neuron sample lacking repeats was subtracted from the corresponding test samples.

Measurement of poly(GA). Neuron pellets were thawed on ice in approximately 120 μl of lysis buffer (1X TBS, pH 7.4, 1 mM EDTA, 1% Triton X-100) with protease inhibitor cocktail (cOmplete, Sigma), vortexed, and incubated at 4°C for 15 min to fully lyse the pellet. Lysed cells were centrifuged at 14,000 RPM for 20 min at 4°C. Total protein concentration of the remaining

supernatant was determined with the BCA protein assay (Thermo Scientific). Poly(GA) content was measured with a Meso Scale Discovery sandwich immunoassay. In this assay, the human/murine chimeric form of anti-GA antibody chGA3 is used as capture antibody, and human anti-GA antibody GA4 along with a SULFO-tag anti-human secondary antibody is used for detection. Poly(GA) concentrations were interpolated from the standard curve by using 60X-GA expressed in HEK 293 cells and were expressed as ng/mg total protein. For background correction, values from a no-repeats neuron sample was subtracted from the corresponding test samples (all values equal to or slightly lower than that were considered zero).

C. References

Choi SY, Lopez-Gonzalez R, Krishnan G, Phillips HL, Li AN, Seeley WW et al. (2019) C9ORF72-ALS/FTD-associated poly(GR) binds Atp5a1 and compromises mitochondrial function in vivo. Nat Neurosci 22:851–862

Lopez-Gonzalez R, Yang D, Pribadi M, Kim TS, Krishnan G, Choi SY et al. (2019) Partial inhibition of the overactivated Ku80-dependent DNA repair pathway rescues neurodegeneration in *C9ORF72*-ALS/FTD. Proc Natl Acad Sci U S A. 116:9628–9633

Lopez-Gonzalez R, Lu Y, Gendron TF, Karydas A, Tran H, Yang D, et al. (2016) Poly(GR) in *C9ORF72*-related ALS/FTD compromises mitochondrial function and increases oxidative stress and DNA damage in iPSC-derived motor neurons Neuron 92:383–391

D. Author contributions

GK, YZ and SA performed experiments. YG and MWK measured poly(GA) levels. FBG and SA analyzed data and supervised the project. SA wrote the manuscript with inputs from all the authors.
